# Severe Sub-Acromial Bursitis with Rice Bodies in a Patient with Rheumatoid Arthritis: A Case Report and Review of Literature

**DOI:** 10.5704/MOJ.1807.010

**Published:** 2018-07

**Authors:** PS Joshi

**Affiliations:** Department of Orthopaedics, Atharva Orthopaedic Superspeciality Hospital, Ahmedabad, India

**Keywords:** rice bodies, sub-acromial bursa, bursitis, rheumatoidarthritis

## Abstract

Multiple rice body formation is a rare presentation of chronic sub-acromial bursitis secondary to extensive underlying rheumatoid arthritis, sero-negative inflammatory arthritis or tuberculous joints. Although there is usually an accompanying inflammatory arthropathy, it can occur in the absence of any underlying systemic disorder. We report a case of five years old neglected rice body deposition in shoulder in a 54-years old lady diagnosed to be having rheumatoid arthritis. After initial investigations, arthroscopic removal of rice bodies with bursa excision relieved the symptoms. The underlying rheumatic condition continued its course, generally sparing the involved shoulder joint. We suggest thorough examination for systemic rheumatologic disease in patients diagnosed with such lesions.

## Introduction

Multiple rice bodies in joints or bursae may be the presenting sign of an extensive underlying rheumatoid or seronegative arthropathy. Less commonly, rice bodies can be seen in periarticular bursae or at the site of tendon or ligament insertion^1^. Involvement of sub-acromial bursa, a rare occurrence in itself, in particular can reach impressive dimensions before becoming clinically evident because of spacious sub-deltoid space. Irritation of surrounding structures such as rotator cuff, acromioclavicular joint and deltoid muscle leads to diffuse painful restriction of shoulder movements. Not commonly, a sub-acromial bursa containing large numbers of rice bodies without extension into the glenohumeral joint may be the presenting sign of a severe rheumatoid condition. We present a rare case of rice body deposition, neglected over a period of five years, in the sub-acromial space in a 54-year old lady with rheumatoid arthritis.

## Case Report

A 54-year old female presented with complaints of painful swelling with restricted movements of right shoulder for past five years which had worsened over the last one year. She was a known case of rheumatoid arthritis diagnosed ten years ago. Five years back she had noticed painful fullness around her right shoulder. Size of the swelling had increased over the period of five years. She had felt her shoulder becoming stiffer with increase in the size of the swelling. On examination there was a well demarcated, firm with smooth surface, non-fluctuant and immobile swelling located at the right sub-deltoid space, with purplish discoloration of the overlying skin. Right shoulder movements were painfully restricted. Sub-acromial impingement signs were present.

**Fig. 1:**
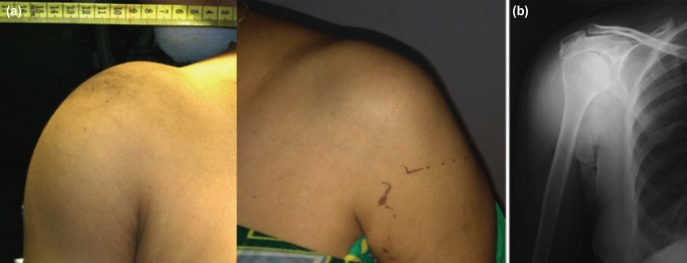
(a) Clinical photographs of sub-deltoid swelling. (b) Plain radiograph of right shoulder with normal glenohumeral articulation and sub-deltoid soft tissue shadow.

**Fig. 2:**
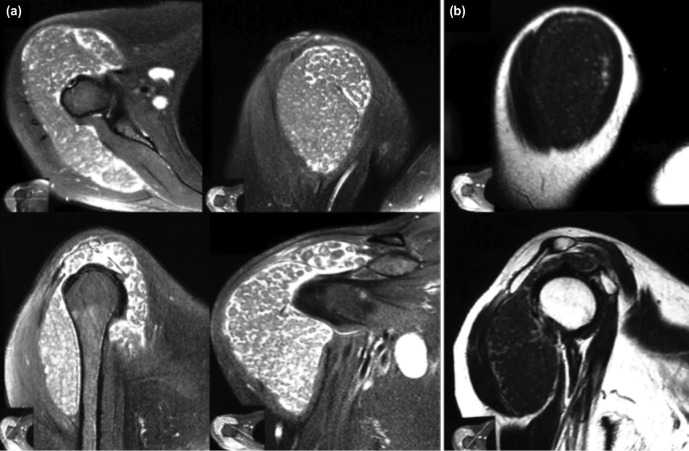
(a) MRI of right shoulder axial, coronal and sagittal T1 weighted images showing multiple iso-intense rice bodies. (b) MRI of right shoulder T2 weighted images showing hypo-intense rice bodies.

Her ESR was 104, Rheumatoid factor 121.2 IU/ml and Uric acid 4.9 mg/dl. Plain radiograph of shoulder showed normal glenohumeral articulation with sub-deltoid homogenous soft tissue shadow without calcification (Fig. 1). Ultrasound suggested diffusely enechoic soft tissue mass surrounding the shoulder joint. MRI suggested marked distension of the sub-acromial/sub-deltoid bursa but more of sub-deltoid bursa, approximately 3cm deep all around. The bursal lumen was filled with multiple loose bodies ranging from 1 to 7mm in size. Loose bodies show iso-intense signal on T1 weighted images and hypo-intense on T2 weighted images. Rotator cuff and glenohumeral joint were normal. This MRI picture was suggestive of synovial origin lesions of arthritis (Fig. 2).

The patient was scheduled for arthroscopic loose body excision and sub-acromial bursectomy. She was positioned in beach chair position under general anaesthesia. Standard posterior, anterior and lateral portals were used for arthroscopy. Diagnostic arthroscopy revealed normal glenohumeral articulation without communication of sub-acromial sub-deltoid bursa with the joint. Multiple shiny white rice bodies of different sizes were observed occupying the sub-acromial space and extending into the anterior, lateral and posterior sub-deltoid space. Rotator cuff muscles were normal. Synovial fluid was submitted for fluid evaluation, Gram stain, Ziehl-Neelson stain, Tuberculosis Polymerase Chain Reaction (TB-PCR) and bacterial culture. Tissue sample from the bursa and rice bodies were sent for histopathological evaluation. All rice bodies were removed through 8mm arthroscopic cannula, placed in different portals sequentially, and attached to a suction device. Residual rice bodies were removed manually with an arthroscopic grasper. Sub-acromial bursa was excised and acromioplasty was performed.

**Fig. 3:**
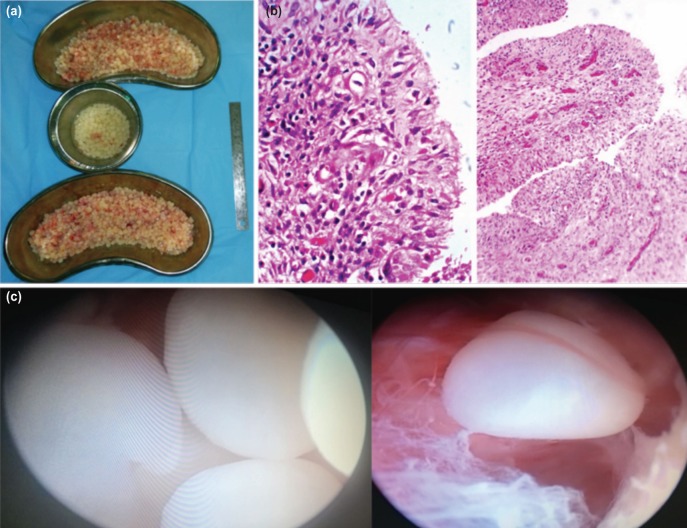
(a) Rice bodies-gross specimen. (b) Rice bodies-microscopic histology consisting of dense fibrin deposition with fibroblast and mono nuclear cells (Hematoxylin-eosin stain, magnification × 200 & x 10). (c) Arthroscopic image of multiple rice bodies in subdeltoid space.

She was started on range of motion exercises of shoulder from first post-operative day. TB-PCR of synovial fluid was negative for tuberculosis. Culture of synovial fluid did not grow any organism. On histopathological examination the bursal tissue showed hyperplastic synovial lining with marked lymphoplasmacytic inflammatory infiltrates, few neutrophils with vascular proliferation of stroma without granulomatous reaction. The rice bodies were composed of dense fibrin deposition with fibroblast and mononuclear cells (Fig. 3). She was started on disease-modifying antirheumatic drugs after suture removal at 12th post-operative day. The pre-operative painful swelling with restriction of movements markedly improved after surgery. At one year post-operative follow-up, there was no recurrence of shoulder swelling, with excellent range of motion.

## Discussion

Rice bodies were first described in tuberculosis joints by Riese in 18961. Rice body formation is usually associated with rheumatoid arthritis, seronegative inflammatory arthritis and tuberculous joints. Occurrence of rice bodies is unrelated to disease duration, severity or associated radiographic change^2^. Rheumatoid involvement of shoulder joint is very rare, especially with association of rice bodies; as seen in our case. Isolated sub-deltoid bursitis associated with massive shoulder swelling secondary to rheumatoid arthritis was described by Palmer, Huston *et al* and Thevenon *et al* in 1969, 1978 and 1987 respectively^3-5^.

The pathogenesis of rice body formation is unclear. Rice bodies usually form secondary to nonspecific response to synovial inflammation. Some authors suggest that they arise from micro-infarcted synovium leading to synovial shedding and subsequent encasement by fibrin derived from synovial fluid. Popert *et al* propose that early rice bodies are formed in synovial fluid independently of synovial elements and progressively enlarge with aggregation of fibrin^2^. On histological examination, they consist of an inner amorphous core of acidophilic material surrounded by collagen and fibrin. Some contain a core of collagen with a mantle of fibrin while others contain only fibrin. The collagen generally consists of types I, III, and V in a proportion of 40-40-20, very similar to that found in synovial membranes.

The main differential diagnoses of multiple rice bodies in sub-deltoid and sub-acromial space are synovial chondromatosis, tuberculosis, synovial ostechondromatosis and pigmented villonodular synovitis. The MRI characteristics of rice bodies have been well documented, with well-defined nodules of intermediate signal on T1-weighted images and relatively low signal on T2-weighted sequences. In contrast, the nodules in synovial chondromatosis will appear high in signal on T2-weighted sequences because of the presence of cartilage component. Tubercular rice bodies are easily differentiated from other condition because of involvement of gleno-humoral joint with significant destruction of joint apart from classical clinical symptoms of tuberculosis. Characteristic histopathological picture of caseating granuloma confirms the diagnosis. While synovial osteochondromatosis of the subacromial bursa is rare, the typical presence of soft tissue ossification on radiography should allow differentiation from multiple rice body formation. Pigmented villonodular synovitis can be differentiated from rice bodies by foci of signal voids reflecting hemosiderosis deposition and lack of susceptibility artifact on gradient echo sequences, which is not seen with rice bodies.

Involvement of sub-acromial and sub-deltoid spaces with rice bodies has potential to cause massive swelling around the shoulder, considering the spacious nature of the subdeltoid area. In cases with severe swelling, possibility of benign or malignant neoplasms must be ruled out. Communication between the bursa and the shoulder joint is usually not the case, although a sub-acromial collection of synovial fluid from joint caused by transgression of the rotator cuff is expected. Arthroscopic resection of the bursa and rice bodies with medical treatment for rheumatoid arthritis provides satisfactory results in symptomatic patients.

## Conflict of Interest

The authors declare no conflicts of interest.
